# Safety Assessment of *Lactobacillus helveticus* KLDS1.8701 Based on Whole Genome Sequencing and Oral Toxicity Studies

**DOI:** 10.3390/toxins9100301

**Published:** 2017-09-24

**Authors:** Bailiang Li, Da Jin, Smith Etareri Evivie, Na Li, Fenfen Yan, Li Zhao, Fei Liu, Guicheng Huo

**Affiliations:** 1 Key Laboratory of Dairy Science, Ministry of Education, Northeast Agricultural University, Harbin 150030, China; 15846092362@163.com (B.L.); 18846420263@163.com (D.J.); besta_intercom@yahoo.com (S.E.E.); lena930313@126.com (N.L.); yffhouse6621@163.com (F.Y.); 18603667208@163.com (L.Z.); 2 Food College, Northeast Agricultural University, Harbin 150030, China; 3 Food Science and Nutrition Unit, Department of Animal Science, Faculty of Agriculture, University of Benin, PMB 1154, Benin City 300001, Nigeria

**Keywords:** probiotics, *Lactobacillus helveticus*, safety assessment, genomics, oral toxicity study, cecal microbiota

## Abstract

*Lactobacillus helveticus* KLDS1.8701 isolated from Chinese traditional fermented dairy product has been shown earlier to possess probiotic potentials but it is important to evaluate its safety in view of its possible use as a probiotic. The aim of the present study is to critically assess the safety of *L. helveticus* KLDS1.8701 through multiple perspectives. The complete genome of *L. helveticus* KLDS1.8701 was sequenced to mine for safety-associated genes. The minimum inhibitory concentrations of 15 antimicrobials and the adverse metabolites were determined. Standard acute oral and subacute toxicity studies were conducted in rats. The results in silico disclosed that the genome of *L. helveticus* KLDS1.8701 carries no transferable antibiotic resistance genes, no virulence factors and only 3 genes related to adverse metabolites. In vitro results showed that *L. helveticus* KLDS1.8701 was resistant against 6 antimicrobials and did not raise safety concerns about biogenic amine, D-lactic acid and nitroreductase. The results in vivo revealed that no adverse effects on experimental rats were observed in the oral toxicity tests. Overall, findings from this study suggest that *L. helveticus* KLDS1.8701 is safe and can be used as a potential probiotic for human consumption.

## 1. Introduction

Probiotics are defined as live microorganisms that confer a health effect on the host, when consumed in adequate amounts [1]. *Lactobacillus helveticus* is an important industrial thermophilic starter and also is used as a probiotic with many direct and indirect health-promoting properties, including the antagonism of pathogens, the modulation of host immune responses, the impact on the composition of the intestinal microbiota and the reduction the blood pressure, allergens and toxic compounds [2]. Members of the genera *Lactobacillus* used as probiotics are “Generally Regarded As Safe” (GRAS). *L. helveticus* strains are assigned to the Qualified Presumption of Safety (QPS) group by the European Food Safety Authority (EFSA) guidelines, as they are a defined taxonomic group and rarely raise safety concerns with a long history of apparent safe use [3]. However, some cases of *Lactobacillus* associated infections have been reported [4,5,6], thus, a careful study of systematically relevant safety aspects for every novel probiotic strain is necessary. With the exception of traditional oral toxicity tests [7], multidisciplinary approaches are needed for the comprehensive safety assessment of probiotic strains. Genomics as one of the important approaches that plays an increasing role in assessing the desired and undesired effects of microorganisms [8]. Genetic stability, antibiotic resistance genes, virulence factors and genes related to hazardous metabolites can be investigated based on genome sequence for safety assessment of probiotic strains. Similar analyses have been described in the safety assessment of *Lactobacillus plantarum* JDM1 [9], *Bifidobacterium longum* JDM301 [10] and *L. helveticus* MTCC 5463 [11]. Although the genotype and phenotype are generally discrepant, a combination of genomic analysis with phenotypic tests of selected findings is highly effective. Thus, the safety assessments of probiotics should take into consideration the genomic data, phenotypic assays and performance in oral toxicity studies.

*L. helveticus* KLDS1.8701 evaluated in this study was isolated from traditional fermented dairy product in China. Previous in vitro and in vivo studies demonstrated that *L. helveticus* KLDS1.8701 owns several potential probiotic functions, such as the capacity to effectively alleviate diarrhea in mice via modulation of intestinal microflora and improve the function of immune system [12]; the potential to resist artificial gastric juice, intestinal juice, bile salts and adhere to Caco-2 cells [12]; the antimicrobial activity against four food-borne pathogens: *Listeria monocytogenes*, *Salmonella Typhimurium*, *Staphylococcus aureus*, *Escherichia coli* [13]; the capacity to inhibit *Penicillium sp.* and extend the shelf-life of fermented soybean milk [14]. The complete genome sequence of *L. helveticus* KLDS1.8701 was performed and genetic basis with probiotic properties were mined in our previous report [15]. In order to exploit this strain as potential probiotic or bioprotective adjunct culture, the safety assessment of this strain must be guaranteed and performed to avoid raising adverse effects and harmful questions. The aim of the present study is to critically assess the safety of *L. helveticus* KLDS1.8701 based on genetic insight from the genomic data, knowledge of phenotypic assays and performance in oral toxicity studies.

## 2. Results

### 2.1. Taxonomic Identification and Mobilomes

The top-hit information from EzTaxon suggested that the strain KLDS1.8701 shares 99.93% similarity (16S rRNA gene sequence) with *L. helveticus* DSM 20075T, the value of ANI was 99.29% between the strain KLDS1.8701 and *L. helveticus* CNRZ 32. These were also accordant with the phylogenetic trees based on 16S rRNA gene sequence (Figure S1) and indicated that the strain KLDS1.8701 belongs to species *L. helveticus*.

Mobile genetic elements such as conjugative plasmids, IS elements and bacteriophages are supposed to be determinant force of horizontal gene transfer (HGT). Only a plasmid that does not encode any genes related to risk factors was detected in *L. helveticus* KLDS1.8701. *L. helveticus* KLDS1.8701 also encompasses 122 IS elements, 75 of which encode pseudo transposes. A questionable and two incomplete prophage regions were identified in the genome of *L. helveticus* KLDS1.8701 (Table S1), which show differences in GC content compared to the average value of *L. helveticus* KLDS1.8701 genome (36.89%).

Three separate CRISPR loci (Table S2) and 9 CRISPR associated sequence (cas) genes were found in the genome of *L. helveticus* KLDS1.8701 by the CRISPRfinder and searching against the local cas bank, this number are relatively higher than other complete sequenced *L*. *helveticus*. Furthermore, other phage immune system, such as restriction modification (R/M) system was observed in the genome (Table S3).

### 2.2. Antibiotic Resistance and Related Determinants

The antimicrobial susceptibility of *L. helveticus* KLDS1.8701 was determined by the MIC compared with the breakpoints of *L. helveticus* in the EFSA and EUC guidelines (Table 1). *L. helveticus* KLDS1.8701 was resistant to six antimicrobials, chloramphenicol, kanamycin, clindamycin, ciprofloxacin, dalfopristinan and trimethoprim but showed sensitivity to other tested antimicrobials, ampicillin, vancomycin, gentamicin, streptomycin, erythromycin, tetracycline, neomycin, linezolid and rifampicin.

To better understand the genetic elements of the antimicrobial susceptibility, the genome was searched to the CARD by BLAST (E < e^−2^, coverage > 70% and similarity > 30%) for antibiotic resistance genes. The result of analysis in silico identified 50 putative antibiotic resistance genes in the genome of *L*. *helveticus* KLDS1.8701, which included genes related to antibiotic efflux pump complex (22), genes responsible for resistance to elfamycin (1), polymyxin (2), isoniazid (2), mupirocin (2), fosfomycin (1), vancomycin (8), beta-lactam antibiotics (1), aminoglycoside (1), lincosamide (5), fluoroquinolone (3), dalfopristin (1), trimethoprim (1) as shown in Table S4. Remarkable matches with genes that likely encode aminoglycoside, lincosamide, fluoroquinolone, dalfopristin and trimethoprim resistance could account for the phenotypic resistance to kanamycin, clindamycin, ciprofloxacin, dalfopristin and trimethoprim, respectively. No genes encoding CAT responsible for chloramphenicol resistance were identified in the genome, when compared with the CARD database. There were no matches when gene sequences encoding Cfr that relates to modify the target of chloramphenicol were additionally searched against the genome by BLAST. At this point, a search for multidrug transporters of MFS was conducted. Two genes encoding MFS transporters (HUO_RS03540 and HUO_RS09200) were characterized in the genome, which can export phenicols from the bacterial cell and impart chloramphenicol resistance to *L*. *helveticus* KLDS1.8701. Moreover, no mobile elements such as transposases, prophages and IS were found in the flanking regions of the antibiotic resistance genes, implying these genes could not spread to commensal bacteria in humans, or to food-borne pathogenic bacteria.

### 2.3. Putatively Adverse Metabolites and Associated Genes

Assessment of deleterious metabolic activities has been indicated as safety consideration by the guidelines for probiotics [1]. The genes related to adverse metabolites were identified by searching against the genome. No genes encoding β-glucosidase, β-glucuronidase, arylsulphatase and azoreductase were found in *L. helveticus* KLDS1.8701 genome. The genes encoding nitroreductase were HUO_RS07380 and HUO_RS08970 (pseudogene). d-lactate dehydrogenase (HUO_RS01270) was also found in the genome. However, the phenotypic results showed that the nitroreductase was not detected in the fermented supernatant and the optical purity of the produced lactic acid by the assay kit was estimated to be 98.5% l-lactic acid using l/d-lactic acid enzymatic test kit, implying that the production of d-lactic acid may be too little to be considered hazardous to consumers.

Histamine, tyramine, phenylethylamine, and cadaverine are produced by a one-step decarboxylation reaction with their respective active amino acid decarboxylase enzymes, genes encoding these enzymes were absent in the genome of *L. helveticus* KLDS1.8701, those genes encoding the conversion of arginine into putrescine (arginine deiminase, HUO_RS05500; ornithine carbamoyltransferase, HUO_RS05505; carbamate kinase, HUO_RS05510) existed as pseudogenes, leading to a nonfunctional pathway, genes encoding the pathway from agmatine to putrescine were not found in the genome of *L. helveticus* KLDS1.8701. HUO_RS06750 (pseudogene) and HUO_RS06750 encode for ornithine decarboxylase, which catalyze the biosynthesis of the putrescine directly from ornithine, furthermore, the strain carried a cluster for spermidine/purescine ABC transporter (HUO_RS08405 to HUO_RS08420). It is likely that these genes endow the potential of putrescine biosynthesis to the strain. However, the results from HPLC revealed that biogenic amines were not produced by *L. helveticus* KLDS1.8701.

### 2.4. Putative Virulence Factors

The result of virulence factors (Table S5) showed that 12 genes associated with virulence factors were identified by searching against the VFDB. These virulence factors were mainly related to chaperonin, adherence, transcriptional regulation and carbohydrate synthesis, which could not confer virulent properties on this strain. No genes encoding invasion or toxin, which are actually offensive virulence factors, were found in the genome of *L. helveticus* KLDS1.8701.

### 2.5. Acute Oral Toxicity Study

The results of the acute oral treatment of rats showed that signs of toxicity or mortality were not caused in any of the animals by a single dose of 6 × 10^10^ CFU *L. helveticus* KLDS1.8701/kg BW through the oral route. There were no changes in general behavior or in physical activity during the 14 days of observation, body weight change and feed consumption of treatment were not significantly different with the control group. Regarding necropsy, no treatment-related pathological changes of all internal organs were detected by macroscopic observations. In general, there were no abnormalities in rats received *L. helveticus* KLDS1.8701 in the acute oral toxicity study (data not shown).

### 2.6. Subacute Oral Toxicity Study

#### 2.6.1. Clinical Observations

There were no abnormal clinical observations in any of the animals throughout the 28-day experimental period. The results of body weight gain, daily food consumption and food efficiency of both sexes are presented in Table S6. In body weight gain, daily food consumption and food efficiency, there were no treatment-related effects or statistically significant differences of both genders between the control and the experimental groups.

#### 2.6.2. Gross Necropsy and Histopathological Examination

**Figure 1:**
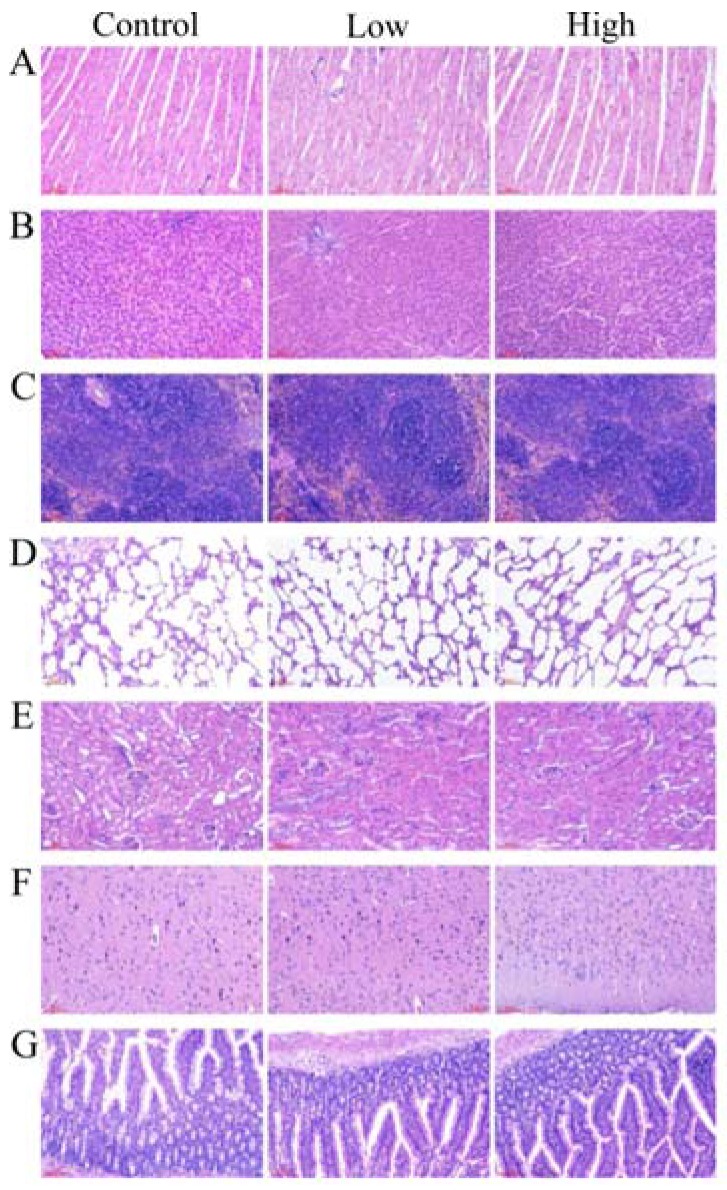
Representative photomicrographs of organs of rats after oral administration of *L. helveticus* KLDS1.8701 for 28 days. Control, sterile normal saline; Low, 1 × 10^9^ CFU of *L. helveticus* KLDS1.8701/kg BW; High, 1 × 10^10^ CFU of *L. helveticus* KLDS1.8701/kg BW; (**A**) heart, (**B**) liver, (**C**) spleen, (**D**) lung, (**E**) kidney, (**F**) brain, (**G**) jejunum. Bar = 50 μm.

No gross pathological findings were detected in rats of all the groups. The relative organ weights were similar in the control and the experimental groups (Table S7), statistical analysis showed that there was no significant difference in the relative organ weights of the experimental groups when compared to the control group. As shown in Figure 1, a single-blind histopathology in all the groups revealed that no histopathological abnormalities were related to *L. helveticus* KLDS1.8701 administration. There were no signs of inflammation, degeneration or necrosis of organs in the control or experimental groups of both genders as determined by the light micrographs of organs, which include heart, liver, spleen, lung, kidney, brain and jejunum of rats.

#### 2.6.3. Hematology and Serum Biochemistry Analysis

The hematological parameters showed that there were no significant alterations of both genders between the control and the experimental groups (Table 2). The results of the serum biochemistry parameters are shown in Table 3. Repeated-dose subacute oral administration of *L. helveticus* KLDS1.8701 also did not cause any significant changes in the serum biochemistry parameters compared with the control group, which are used as biochemical markers of organ damages, the results of this part revealed that all hematological and serum biochemical parameters were situated within the reference ranges.

#### 2.6.4. Cecal Microbiota and Harmful Bacterial Enzymes

**Figure 2:**
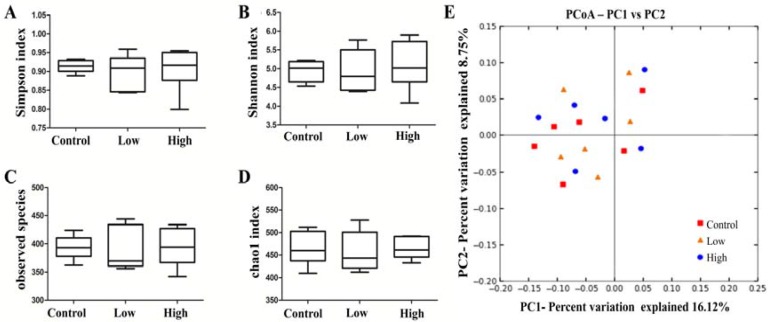
Cecal microbiota α-diversity and β-diversity in male rats after oral administration of *L. helveticus* KLDS1.8701 for 28 days. Control, sterile normal saline; Low, 1 × 10^9^ CFU of *L. helveticus* KLDS1.8701/kg BW; High, 1 × 10^10^ CFU of *L. helveticus* KLDS1.8701/kg BW. (**A**) Simpson index, (**B**) Shannon index, (**C**) observed species, (**D**) Chao1 index, (**E**) Principal coordinates analysis (PCoA) of unweighted UniFrac distances.

**Figure 3:**
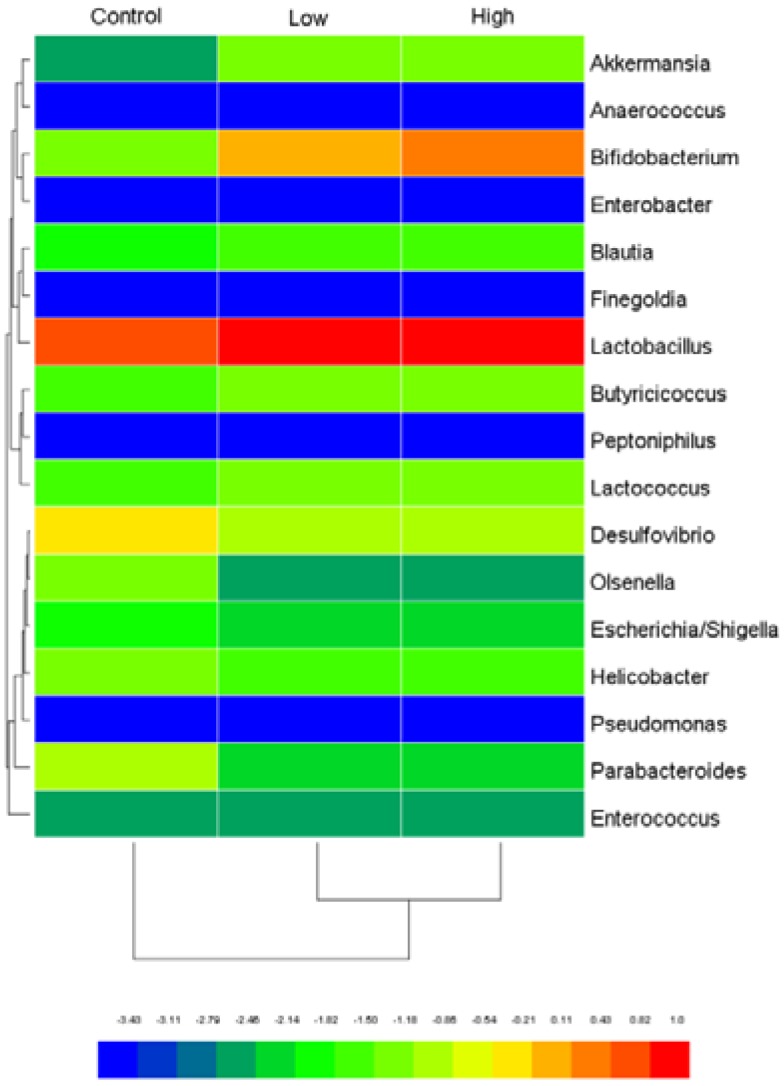
Heat map of safety-related genus in male rats after oral administration of *L. helveticus* KLDS1.8701 for 28 days. Control, sterile normal saline; Low, 1 × 10^9^ CFU of *L. helveticus* KLDS1.8701/kg BW; High, 1 × 10^10^ CFU of *L. helveticus* KLDS1.8701/kg BW.

The effect of *L. helveticus* KLDS1.8701 on cecal microbiota diversity was evaluated by α-diversity and β-diversity. No significant differences in α-diversity, including Simpson index, Chao1 index, Shannon index and observed species (Figure 2A–D), were observed among all the groups in male rats, indicating that *L*. *helveticus* KLDS1.8701 administration did not influence cecal microbiota diversity and richness of healthy male rats. To confirm this result, the β-diversity was determined by the principal coordinate analysis (PCoA) to explore the microbiota similarities among all the groups in male rats. As shown in the figure from the unweighted UniFrac (Figure 2E), the majority of the microbiota structures among all the groups in male rats were very similar. In order to further study the changes in the safety-related genera, the heat map was created from selected genera containing probiotics and opportunistic pathogens (Figure 3), in these genera, *Bifidobacterium* and *Lactobacillus* were predominant compared to *Akkermansia*, *Escherichia/Shigella*, *Desulfovibrio*, *Helicobacter*, *Olsenella*, *Parabacteroides*, *Blautia*, *Enterococcus*, *Butyricicoccus* and *Lactococcus*, however, *Anaerococcus*, *Enterobacter*, *Finegoldia*, *Peptoniphilus* and *Pseudomonas* were not inhabited in these three groups. Almost all genera fluctuated between groups treated with *L*. *helveticus* KLDS1.8701 and the control group, the groups treated with *L*. *helveticus* KLDS1.8701 in male rats showed an increase in genera containing probiotics, such as *Bifidobacterium*, *Akkermansia* and *Lactobacillus* and a decrease in genera containing opportunistic pathogens, such as *Escherichia/Shigella*, *Desulfovibrio*, *Helicobacter*, Olsenella and *Parabacteroides* when compared with the control group. Particularly, *Bifidobacterium* was remarkably increased in the high dose group, whereas the low dose group showed a less pronounced effect on this genus. In addition, the relative abundances of *Blautia*, *Enterococcus*, *Butyricicoccus* and *Lactococcus* in high and low dose groups were not different with the control group. Based on these analyses, repeated-dose subacute oral administration of *L*. *helveticus* KLDS1.8701 could increase certain genera containing probiotics and decrease certain genera containing opportunistic pathogens in the cecal microbiota, implying the benefit of this strain. Similar results from all the groups in female rats were obtained as well (data not shown).

The effect of the *L. helveticus* KLDS1.8701 on the harmful cecal bacterial enzymes (β-glucosidase and β-glucuronidase) was evaluated and the results are shown in Table S8. The concentrations of β-glucuronidase in the cecal contents of both the experimental groups were significantly lower (*P* < 0.05) than the control group. The activities of β-glucosidase in the cecal contents of both the experimental groups showed non-significant differences from the control group.

## 3. Discussion

There has been increased interest in the use *Lactobacillus* as probiotic ingredient in foods and drinks to improve the health of consumers. *L. helveticus* KLDS1.8701 was isolated from Chinese traditional fermented dairy product and its potential probiotic properties were evaluated in our previous study. It thus becomes needful to evaluate the safety of this strain before it uses as a novel commercial probiotic [1]. In this study, the indepth safety assessment of *L. helveticus* KLDS1.8701 was performed using multiple methods. The precise taxonomic characterization is the first step in the safety evaluation for tracking the applied standards (e.g., EUC and EFSA) of the strain. The 16S rRNA sequence-based division into higher taxa is currently the most widely used classification system for prokaryotes. However, the sequence of 16S rRNA gene is too conserved to distinguish between closely related species [16]. ANI has been proposed as the best alternative for a gold standard for prokaryotic species circumscriptions at the genomic level [17]. Our strain was identified as *L. helveticus* using ANI and the phylogenetic trees based on the 16S rRNA gene.

Genomic stability is a key factor to keep attributions of probiotic. *L. helveticus* KLDS1.8701 contains only a plasmid without any risk factors. However, IS elements are generally regarded as active participators in genomic rearrangement, contributing to the variant generation of genetic diversity [18]. Our results show that *L. helveticus* KLDS1.8701 harbors 122 copies of IS elements. Similarly, the 154 and 213 IS elements are loaded to the genome of *L. helveticus* MTCC 5463 [11] and *L. helveticus* DPC 4571 [18], respectively, which have been demonstrated exceptional stability. CRISPR/Cas system and R/M system are the important barriers to resist to HGT. *L. helveticus* KLDS1.8701 carries CRISPR/Cas system and Type I R/M system, which are in line with no intact prophage and make a contribution to keep the genome stable.

*Lactobacillus* may act as reservoir of antibiotic resistance genes, which can spread to commensal bacteria in humans or animals, or to food-associated pathogens [19]. When microbial strains used in food applications show resistance to clinically relevant antimicrobials, the antibiotic resistance genes and probability of occurrence of HGT must be evaluated [20]. The present study has shown that *L*. *helveticus* KLDS1.8701 was resistant against 6 antimicrobials and equipped 50 putative antibiotic resistance genes, most of them are genes related to antibiotic efflux pump complex. As indicated in literatures [9,10], there are discrepancies between the phenotype and the genotype in antibiotic resistance. For example, *L*. *helveticus* KLDS1.8701 was sensitive to ampicillin but contained the beta-lactam antibiotic resistance gene, it could be explained by inhibition by its detected repressor BlaI (HUO_RS00790), which regulates resistance to beta-lactams. *L*. *helveticus* KLDS1.8701 was also sensitive to very low dose of vancomycin, despite the determinant genes of vancomycin were characterized in the genome. This result is in consonance with *B*. *longum* JDM30 and *L*. *helveticus* IMAU60226 [10,21]. It is possible that these genes were not sufficiently expressed or only induced under certain circumstances, including environmental stimulus or surrounding signals. Additional studies will be needed to determine this discrepancy, because there are many other reasons for the discrepancies between the phenotype and the genotype in antibiotic resistance, it can also be assumed that genes retrieved by in silico analysis were modified after transcription or pseudogenes or only partially similar to known resistance genes, but do not represent a harmful trait of this bacterium. Interestingly, no chloramphenicol resistance genes were found in the genome of *L*. *helveticus* KLDS1.8701 when compared with the CARD database, but this strain was highly resistant to chloramphenicol (Table 1), which was in agreement with a previous report on *L. helveticus* NNIE [22]. Chloramphenicol binding sites cluster at the peptidyl-transferase centre (PTC) of the ribosome large (50S) subunit, which is composed by 34 ribosomal proteins and 5S and 23S rRNA genes, and chloramphenicol resistance mechanisms include chloramphenicol modification, mutation and modification of the target, and multidrug-resistance efflux pumps [23]. CAT can detoxify the antibiotic chloramphenicol by attaching an acetyl group to chloramphenicol, which prevents chloramphenicol from binding to ribosomes [24]. Cfr can monomethylate the C8 atom of the 23S rRNA nucleotide A2503 (located in the PTC), conferring resistance to chloramphenicol [25]. MFS transporters can export phenicols from the bacterial cell [23]. After ruling out the presence of the genes encoding CAT and Cfr as being responsible for the exceptionally high resistance to chloramphenicol in *L*. *helveticus* KLDS1.8701, the reason for chloramphenicol resistance is the attendance of the MFS transporters. Our findings show that the determinant antibiotic resistance genes of *L. helveticus* KLDS1.8701 could not be transferred to pathogens due to the absence of mobile elements in their flank. Furthermore, the attendance of CRISPR loci can restrict the dissemination of antibiotic resistance gene by eliminating multiple route of HGT [26].

Higher alimentary intake of biogenic amines have adverse effects on the health of consumers [27], thus, the assessment of this strain’s capacity to produce biogenic amines is required. Although *L. helveticus* KLDS1.8701 has putrescine biosynthesis genes based on genomic data, no biogenic amines were detected by HPLC. *L. helveticus* CNRZ 32 with similar genetic elements does not convert ornithine to putrescine, indicating that the genes are either not functional or ornithine decarboxylase does not possess activity [28]. In this study, a gene encoding d-lactate dehydrogenase was found in the genome of this strain, but it was demonstrated that the production of d-lactate was insufficient to cause the d-lactic acidosis by the optical purity of the produced lactic acid.

Safety assessment in vivo can provide more effective information, because the physiology of rats is similar to the corresponding human condition in many cases [29]. In this study, standard acute oral and subacute toxicity studies in rats were conducted. In the acute oral toxicity study, there was no mortality or signs of toxicity, there were also no changes in weight losses, feed consumption, general behavior or pathology in all rats. In order to further evaluate the safety of *L. helveticus* KLDS1.8701, a 28-day repeated-dose subacute oral toxicity study with two dose levels was performed. Changes in food consumption, body weight and organ weights are considered as indicators of toxic effects of a test material [30]. In this study, there were no significant differences in these aspects between the control and experimental groups. Analysis of hematological and serum biochemical parameters is relevant for risk evaluation, because these values could reflect the problems in organs [31]. No significant differences between the control and experimental groups in terms of hematological and serum biochemical parameters were observed in this study. Histopathology can provide clear clinical advantage, *L. helveticus* KLDS1.8701 intake did not lead to any histopathological abnormalities or changes.

Cecal content is a good material for safety assessment in the subacute oral toxicity study, on one side, the harmful bacterial enzymes were studied, the concentrations of β-glucosidase and β-glucuronidase in the cecal contents were significantly lower and not significantly different, respectively, when compared to the control group. On the other hand, the cecal bacterial populations that play crucial roles in maintaining the host’s health, were studied by sequencing the 16S rRNA gene V4 region. *L*. *helveticus* KLDS1.8701 administration did not influence cecal microbiota diversity and richness of healthy male rats, as no significant differences in α- and β-diversity were detected. Our data are consistent with previous studies which highlighted no marked effects of probiotics on the gut microbiota diversity and richness [32]. To further analyze the special bacteria taxa associated with *L*. *helveticus* KLDS1.8701 administration, the heat map was used to compare the bacterial taxa abundance between the experimental groups and the control group. The high and low dose groups in male rats showed an increase in genera containing probiotics, such as *Bifidobacterium*, *Akkermansia* and *Lactobacillus* compared with the control group. Numerous species belonging to *Lactobacillus* and *Bifidobacterium* have been reported as safe and effective in improving the host’s health for a long history [33]. *A. muciniphila* belonging to *Akkermansia* has been considered as a next-generation probiotic, which can have ameliorative effects on obesity and reverse high fat diet-induced obesity [34]. No changes in *Blautia* and *Butyricicoccus* were observed in different groups, short-chain fatty acids produced by which can act as main fuel for enterocytes and influence diverse cellular functions [35,36]. Differently, an opposite trend was found for genera containing opportunistic pathogens such as *Escherichia/Shigella*, *Helicobacter*, *Desulfovibrio*, *Olsenella* and *Parabacteroides*, which abundances were decreased when compared to the control group. *Helicobacter*, *Escherichia/Shigella* and *Desulfovibrio* are often associated with the pathogenesis of inflammatory bowel disease (IBD) and ulcerative colitis (UC), both of which have deleterious effects on the immune system and enhance intestinal inflammation [37,38]. Some species of *Olsenella* and *Parabacteroides* represent opportunistic pathogens in infectious diseases [39,40]. As a result, these changes can be considered as positive marks due to *L*. *helveticus* KLDS1.8701, which seems to bring healthy benefit to rat by modulating gut microbiota. To our knowledge, we are the first to use this strategy in the subchronic rodent study for safety assessment.

## 4. Conclusions

This study demonstrated that *L. helveticus* KLDS1.8701 does not raise safety concerns about transferable antibiotic resistance genes, virulence factors, production of biogenic amine and D-lactic acid, activity of nitroreductase by the combination phenotypic profiles with genomic data. Furthermore, no adverse effects were observed regarding clinical observations, hematological and serum biochemical parameters, histopathological study, cecal harmful bacterial enzymes or cecal bacterial populations by safety assessment in vivo. The comprehensive results suggest that *L. helveticus* KLDS1.8701 is safe and can be used as a potential probiotic for human consumption.

## 5. Materials and Methods

### 5.1. Bacteria Strain and Growth Condition

*L. helveticus* KLDS1.8701 was isolated from traditional fermented dairy product in China and stored in Key Laboratory of Dairy Science (KLDS), Ministry of Education, China. The strain was anaerobically incubated in deMan Rogosa and Sharpe (MRS) broth at 37 °C for 18 h. The bacterial strain was sub-cultured twice prior to the experiment. The bacteria were harvested by centrifugation (10 min, 5000 × *g*, 4 °C) and the resulting cell pellets were washed three times with ultrapure water and re-suspended at the desired concentration in sterile normal saline.

### 5.2. Genome Sequencing and Taxonomy

The genomic DNA of *L. helveticus* KLDS1.8701 was extracted by the DNeasy Tissue kit (Qiagen, Germany). The details of genome sequencing were described by our previous report [15]. The top-hit strain with *L. helveticus* KLDS1.8701 was identified using the web-based EzTaxon server [41] on the basis of 16S rRNA gene, then the 16S rRNA gene sequence was aligned with those of top-hit strain and related strains by CLUSTAL W. The phylogenetic trees were constructed by MEGA 6.0 software [42] using Neighbour-joining method. Average nucleotide identity (ANI) of two genome sequences was valued by ANI Calculator using the OrthoANIu algorithm [43].

### 5.3. Identification of Safety-Associated Genes in Silico

All annotated genes of *L. helveticus* KLDS1.8701 were additionally mapped against the comprehensive antibiotic resistance database [44] and the virulence factors database [45]. Insertion sequences (IS), clustered regularly interspersed short palindromic repeats (CRISPR) and prophage sequences were identified using ISfinder [46] and the genome annotation, CRISPRFinder [47] and PHASTER [48], respectively. The gene sequences related to the adverse metabolites (amino acid decarboxylases, β-glucosidase, β-glucuronidase, arylsulphatase, azoreductase, nitroreductase and d-lactate dehydrogenase) and chloramphenicol resistance (chloramphenicol acetyl transferase (CAT), 23S rRNA gene methyltransferase (Cfr) and major facilitator superfamily (MFS) transporters) were downloaded from Genbank and searched against the genome by BLASTN. Hits with more than 70% coverage and 30% identity were chosen as the positive results in this study.

### 5.4. Measurement of Antibiotic Resistance Phenotypes

The minimal inhibitory concentrations (MICs) for 15 antimicrobials (ampicillin, vancomycin, tetracycline, chloramphenicol, kanamycin, gentamicin, streptomycin, erythromycin, clindamycin, dalfopristin, linezolid, ciprofloxacin, rifampicin and trimethoprim) of *L. helveticus* KLDS1.8701 were determined by a macro dilution method according to standardized methods [49]. The antimicrobials susceptibility was defined by the reference cut-off value in the EFSA [20] and European Commission (EUC) guidelines [50].

### 5.5. Measurement of Adverse Metabolites

High performance liquid chromatography (HPLC) was employed to detect biogenic amines (tryptamine, 2-phenyl–ethylamine, putrescine, tyramine, cadaverine, histamine, tyramine, spermidine and spermine) production by *L. helveticus* KLDS1.8701. The pretreatment and derivatisation of the samples were performed as outlined by Dadáková et al. and Lorencováet al. [51,52]. HPLC measurements were conducted on Waters Alliance HPLC system (Waters e2695, Milford, MA, USA) consisting of a binary pump and a UV/Vis detector (λ = 245 nm). The separation was achieved using a C-18 column (250 mm × 4.6 mm, 5 μm, SANYO, Osaka, Japan). The specific conditions for separation and detection of biogenic amines are as previously described [53]. Chromatogram of biogenic amines in standard solution was shown in Figure S2. The activity of nitroreductase was measured following the instructions on the assay kit (Nanjing Jiancheng Bioengineering Institute, Nanjing, China). The optical purity of the produced lactic acid was estimated using l/d-lactic acid enzymatic test kit in accordance with the recommendations of the manufacturer (Megazyme, Bray, Ireland).

### 5.6. Animals

Specific Pathogen Free (SPF) Sprague-Dawley rats (6–7 weeks old, male and female) were purchased from Vital River Laboratory Animal Technology Co., Ltd. (Beijing, China) and housed in a room under controlled environmental conditions at 23 ± 2 °C, a relatively humidity of 50 ± 20% and a 12-h light/dark cycle. All the rats were put into the plastic cages for one week of acclimatization to the laboratory conditions before beginning the experiments. Food and water were available ad libitum. The experimental protocols were approved by the Animal Use and Ethics Committee of Northeast Agricultural University (Harbin, China) under the approved protocol number Specific pathogen free rodent management (SRM)-06 (30 10 2011). The care procedures for the animals were implemented following the European Community guidelines (Directive 2010/63/EU).

### 5.7. Experimental Design

#### 5.7.1. Acute Oral Toxicity Study

The acute oral toxicity study was a single-dose toxicity study. It was conducted according to the OECD guideline [54]. Healthy rats of either sex were randomly divided in two groups of six rats each, namely the control group received 1 mL of normal saline, experimental group was treated with 6 × 10^10^ CFU of *L*. *helveticus* KLDS1.8701/kg body weight (BW) in 1 mL of sterile normal saline, which were administered a single dose by oral gavage. The animals were observed for the signs of toxicity for continuous 4 h after inoculation and once daily for 14 days. General behavioral observations recommended by the OECD guideline [54] were made once daily for 14 days. Body weight and feed consumption were recorded. On day 15, all rats were fasted for 16 h and then humanely sacrificed under diethyl ether anesthesia. Macroscopic observation of the animals was performed during necropsy.

#### 5.7.2. Subacute Oral Toxicity Study

The subacute oral toxicity study was performed following the OECD guideline [55]. Healthy rats of either sex were randomly divided in three groups of six rats each. Rats in the control group were administered with 1 mL of sterile normal saline, while rats of low-dose and high-dose groups were treated once daily through intragastric gavage with *L. helveticus* KLDS1.8701 at a dose of 1 × 10^9^ CFU/kg BW (Low) and 1 × 10^10^ CFU/kg BW (High), respectively. Body weights and food consumption were measured weekly, then food efficiency was calculated as a ratio of body weight gain to food consumption and clinical signs of morbidity or mortality were recorded daily throughout the period administration animals.

### 5.8. Hematological and Serum Biochemistry Analyses

At the end of the dosing period, all animals were fasted for 16 h and then humanely sacrificed under diethyl ether anesthesia. Blood samples were collected in heparinized tubes and assessed for red blood cell count, white blood cell count, hemoglobin, platelet count, mean corpuscular volume, mean corpuscular hemoglobin concentration, neutrophils, lymphocytes, monocytes and eosinophils by automatic hematological analyzer (Nihon Kohden, Tokyo, Japan). The serum centrifuged from the blood samples were used for clinical biochemistry measurement by automatic biochemistry analyzer (Toshiba, Tokyo, Japan), the following parameters were tested: aspartate aminotransferase, alanine aminotransferase, alkaline phosphatase, total bilirubin, total protein, albumin, glucose, triglycerides, total cholesterol, high density lipoprotein cholesterol, low density lipoprotein cholesterol, urea, creatinine, sodium, chloride calcium and inorganic phosphorus.

### 5.9. Gross Necropsy and Histopathological Studies

After the sacrifice, organs, including heart, liver, spleen, lung, kidney, brain, adrenal thymus, testes (male rats), epididymides (male rats), uterus (female rats) and ovary (female rats) were collected from each animal for gross necropsy and organ weights, the relative organ weights were calculated as percentage of organ weights to terminal body weight, then organs (heart, liver, spleen, lung, kidney, brain and jejunum) were dissected out and fixed in 10% neutral formalin for 18 h. Samples were then embedded in paraffin, sliced into 5 μm thickness and stained with hematoxylin-eosin for examination by light microscopy.

### 5.10. Cecal Microbiota and Harmful Bacterial Enzymes

Total microbiota genomic DNA was extracted from cecal contents by a QIAamp DNA stool mini kit (Qiagen, Hilden, Germany). The V4 region of 16S rDNA was amplified with universal primers, sequenced by Illumina Miseq (Illumina, San Diego, CA, USA) and then analyzed using QIIME software (v1.6) [56]. The details of this pipeline were as recently described by Xie et al. [57]. In addition, a heat map was constructed to assess the safety status and the main genera in the different groups studied were *Bifidobacterium*, *Akkermansia*, *Lactobacillus*, *Escherichia/Shigella*, *Desulfovibrio*, *Helicobacter*, *Olsenella*, *Parabacteroides*, *Anaerococcus*, *Enterobacter*, *Blautia*, *Enterococcus*, *Butyricicoccus*, *Lactococcus*, *Finegoldia*, *Peptoniphilus* and *Pseudomonas*. Harmful intestinal bacterial enzymes (β-glucosidase and β-glucuronidase) of cecal contents from the rats were evaluated by the steps as previously described [58].

### 5.11. Statistical Analysisd

All values are expressed as means ± the standard deviation (SD). A minimum of three independent experiments were carried out for each assay. Statistical significance of data comparisons was determined using one-way analysis of variance (ANOVA), followed by Duncan’s Multiple Range test. Values of *P <* 0.05 were considered to be statistically significant.

## Figures and Tables

**Table 1:** Minimum inhibitory concentrations (MICs) of *L. helveticus* KLDS1.8701 towards 15 antimicrobials and the microbiological cut-off values.

Antimicrobials	MIC	Cut-Off Values
Ampicillin	<0.25	1
Vancomycin	1	2
Gentamicin	16	16
Streptomycin	8	16
Kanamycin	>256	16
Erythromycin	<0.25	1
Clindamycin	>256	1
Chloramphenicol	>256	4
Tetracycline	2	4
Dalfopristin	8	4
Linezolid;	2	4
Neomycin	4	32
Ciprofloxacin	16	4
Trimethoprim	64	32
Rifampicin	2	32

**Table 2:** Hematological parameters of male and female rats after oral administration of *L. helveticus* KLDS1.8701 for 28 days.

Treatment	Males			Females		
	Control	Low	High	Control	Low	High
RBC (×10^6^/µL)	7.73 ± 0.42	7.93 ± 0.55	8.26 ± 0.99	7.47 ± 0.45	7.63 ± 0.32	7.64 ± 0.39
WBC (×10^3^/µL)	13.37 ± 1.2	12.10 ± 2.25	10.77 ± 1.34	11.97 ± 1.56	10.63 ± 1.92	13.17 ± 1.95
HGB (g/L)	148.30 ± 6.05	150.93 ± 5.47	149.47 ± 9.05	142.57 ± 2.80	142.3 ± 5.51	141.87 ± 3.02
PLT (×10^3^/µL)	909.23 ± 101.57	920.80 ± 146.94	957.33 ± 193.27	1226.67 ± 213.73	1350.18 ± 156.90	1292.33 ± 237.05
MCV (fL)	56.03 ± 1.50	55.27 ± 2.61	57.00 ± 9.54	55.12 ± 1.28	54.37 ± 1.26	55.27 ± 2.78
MCHC (g/L)	331.43 ± 10.81	333.37 ± 11.15	336.33 ± 9.61	340.83 ± 5.01	340.33 ± 7.23	337.67 ± 8.02
Neutrophils (%)	19.47 ± 2.35	20.80 ± 0.95	21.97 ± 3.24	24.84 ± 4.06	25.73 ± 0.67	21.13 ± 2.90
Lymphocytes (%)	71.43 ± 5.61	72.17 ± 2.20	73.97 ± 1.70	73.73 ± 3.12	75.73 ± 3.17	74.21 ± 6.26
Monocytes (%)	3.83 ± 0.31	3.91 ± 0.44	4.12 ± 0.43	5.17 ± 0.45	4.97 ± 1.16	4.87 ± 0.67
Eosinophils (%)	1.40 ± 0.44	1.13 ± 0.30	1.39 ± 0.47	1.30 ± 0.61	1.23 ± 0.45	1.05 ± 0.13

Values are presented as mean ± standard deviation (*n* = 6). Control, sterile normal saline; Low, 1 × 10^9^ CFU of *L. helveticus* KLDS1.8701/kg BW; High, 1 × 10^10^ CFU of *L. helveticus* KLDS1.8701/kg BW; RBC, red blood cell count; WBC, white blood cell count; HGB, hemoglobin; PLT, platelet count; MCV, mean corpuscular volume; MCHC, mean corpuscular hemoglobin concentration

**Table 3:** Serum biochemical parameters of male and female rats after oral administration of *L. helveticus* KLDS1.8701 for 28 days.

Treatment	Males			Females		
	Control	Low	High	Control	Low	High
AST (U/L)	148.80 ± 5.99	151.67 ± 20.03	148.67 ± 14.05	91.27 ± 8.75	94.67 ± 11.85	93.47 ± 5.08
ALT (U/L)	72.77 ± 8.88	72.56 ± 6.24	75.33 ± 5.69	52.53 ± 7.36	55.13 ± 4.59	53.33 ± 3.79
ALP (U/L)	159.33 ± 9.29	161.67 ± 14.19	168.00 ± 10.54	88.33 ± 8.50	88.67 ± 10.97	92.67 ± 5.51
TBIL (μmol/L)	1.00 ± 0.36	0.89 ± 0.11	1.07 ± 0.25	1.03 ± 0.21	1.01 ± 0.29	1.02 ± 0.10
TP (g/L)	53.33 ± 3.68	53.16 ± 4.54	54.01 ± 6.53	67.77 ± 4.50	68.53 ± 3.26	67.33 ± 7.70
ALB (g/L)	42.13 ± 3.44	38.41 ± 3.61	36.73 ± 4.65	42.93 ± 2.60	43.63 ± 4.40	44.87 ± 4.96
GLU (mmol/L)	5.86 ± 1.60	6.37 ± 0.97	6.60 ± 0.75	8.97 ± 0.87	8.37 ± 0.92	8.65 ± 0.74
TG (mmol/L)	0.48 ± 0.05	0.53 ± 0.12	0.49 ± 0.07	0.51 ± 0.02	0.51 ± 0.05	0.49 ± 0.05
TC (mmol/L)	1.42 ± 0.11	1.47 ± 0.18	1.53 ± 0.35	2.23 ± 0.25	2.17 ± 0.31	2.33 ± 0.57
HDL (mmol/L)	1.68 ± 0.25	1.70 ± 0.02	1.37 ± 0.31	1.77 ± 0.31	1.71 ± 0.18	1.87 ± 0.35
LDL (mmol/L)	0.21 ± 0.09	0.21 ± 0.10	0.15 ± 0.07	0.33 ± 0.08	0.32 ± 0.03	0.31 ± 0.04
UREA (mmol/L)	6.47 ± 1.32	7.03 ± 0.15	6.63 ± 0.61	6.63 ± 0.38	6.47 ± 0.31	6.53 ± 0.67
CRE (μmol/L)	52.70 ± 4.93	56.67 ± 5.86	52.67 ± 5.69	73.17 ± 2.35	72.13 ± 3.75	71.53 ± 3.18
Na (mmol/L)	140.93 ± 2.35	142.01 ± 3.00	143.87 ± 7.96	142.17 ± 3.25	142.07 ± 7.87	144.33 ± 6.03
Cl (mmol/L)	105.40 ± 2.33	100.33 ± 2.52	101.33 ± 7.57	100.67 ± 2.37	98.73 ± 2.28	100.73 ± 3.55
Ca (mmol/L)	2.69 ± 0.13	2.45 ± 0.22	2.55 ± 0.32	2.56 ± 0.07	2.55 ± 0.06	2.54 ± 0.11
P (mmol/L)	3.12 ± 0.30	2.87 ± 0.21	2.83 ± 0.39	2.48 ± 0.07	2.61 ± 0.12	2.52 ± 0.03

Values are presented as mean ± standard deviation (*n* = 6). Control, sterile normal saline; Low, 1 × 10^9^ CFU of *L. helveticus* KLDS1.8701/kg BW; High, 1 × 10^10^ CFU of *L. helveticus* KLDS1.8701/kg BW; AST, aspartate aminotransferase; ALT, alanine aminotransferase; ALP, alkaline phosphatase; TBIL, total bilirubin, TP, total protein; ALB, albumin; GLU, glucose; TG, triglycerides; TC, total cholesterol; HDL, high density lipoprotein cholesterol; LDL, low density lipoprotein cholesterol; UREA, urea; CRE, creatinine; Na, sodium; Cl, chloride; Ca, calcium; P, inorganic phosphorus.

## Supplementary Materials

The following are available online at www.mdpi.com/2072-6651/9/10/301/s1, Figure S1: Neighbour-joining tree based on the 16S rRNA gene sequences of strain KLDS1.8701 and phylogenetically related type strains. Bootstrap values based on 1000 resampled datasets are shown at branch nodes, Figure S2: Typical HPLC chromatograms of biogenic amines in standard solution. 1, tryptamine; 2, 2-phenyl – ethylamine; 3, putrescine; 4, cadaverine; 5, histamine; 6, tyramine; 7, spermidine; 8, spermine, Table S1: General features of the phage regions identified in the *L. helveticus* KLDS1.8701 genome, Table S2: General features of the CRISPR loci identified in the *L. helveticus* KLDS1.8701 genome, Table S3: Putative genes for Type I restriction modification (R/M) system in the *L. helveticus* KLDS1.8701 genome, Table S4: Putative antibiotics resistance genes in the *L. helveticus* KLDS1.8701 genome by searching with the comprehensive antibiotic resistance database (CARD), Table S5: Putative virulence factors in the *L. helveticus* KLDS1.8701 genome by searching with the virulence factor database (VFDB), Table S6: Body weight and food data of male and female rats after oral administration of *L. helveticus* KLDS1.8701 for 28 days, Table S7: Relative organ weights (%) of male and female rats after oral administration of *L. helveticus* KLDS1.8701 for 28 days, Table S8: β-glucosidase and β-glucuronidase activities in the cecal contents of male and female rats after oral administration of *L. helveticus* KLDS1.8701 for 28 days.
