# Supplementary material for: Safety Assessment of Lactobacillus helveticus KLDS1.8701 Based on Whole Genome Sequencing and Oral Toxicity Studies
Source: Toxins (Basel). 2017 Sep 24;9(10):301. doi: 10.3390/toxins9100301 (PMC5666348; doi:10.3390/toxins9100301)
Supplement: Supplementary file 1 [file toxins-09-00301-s001.pdf]

Supplementary Materials: Safety Assessment of Lactobacillus helveticus KLDS1.8701 Based on Whole Genome Sequencing and Oral Toxicity Studies

Bailiang Li, Da Jin, Smith Etareri Evivie, Na Li, Fenfen Yan, Li Zhao, Fei Liu and Guicheng Huo

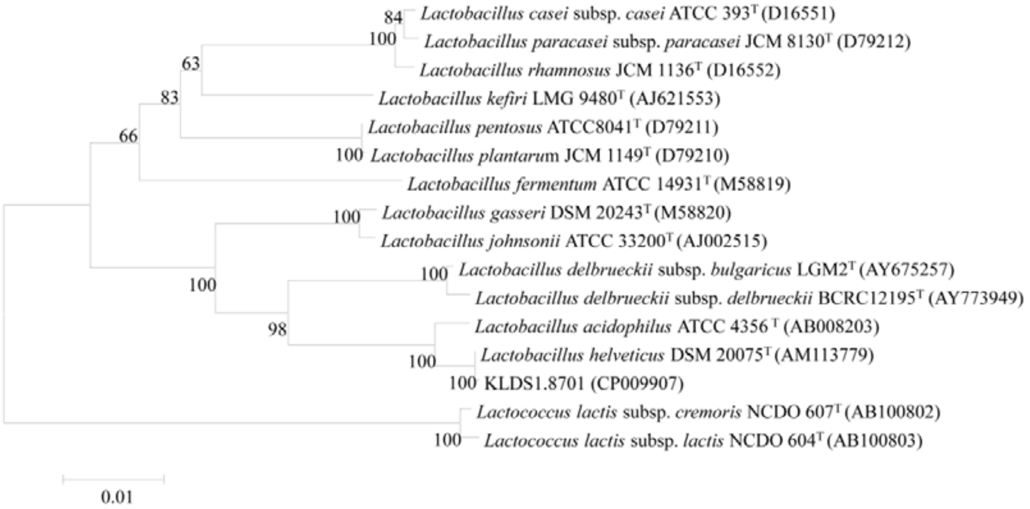

Figure S1 Neighbour-joining tree based on the 16S rRNA gene sequences of strain KLDS1.8701 and phylogenetically related type strains**.** Bootstrap values based on 1000 resampled datasets are shown at branch nodes.

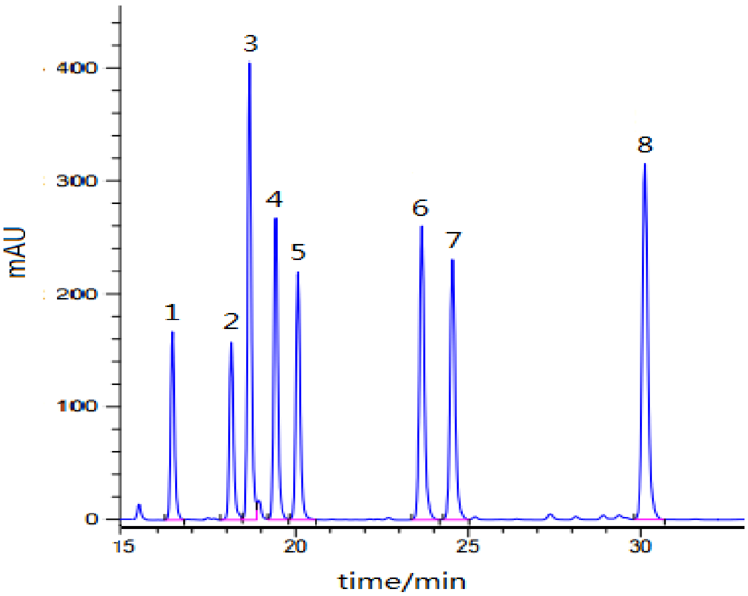

Figure S2 Typical HPLC chromatograms of biogenic amines in standard solution. 1, tryptamine; 2, 2-phenyl – ethylamine; 3, putrescine; 4, cadaverine; 5, histamine; 6, tyramine; 7, spermidine; 8,spermine.

Table S1 General features of the phage regions identified in the *L. helveticus* KLDS1.8701 genome.

Position	Completeness	Total proteins	Most Common Phage	GC percentage
689121-696714	incomplete	13	NC_023719	35.13%
1980240-1988321	incomplete	10	NC_023007	35.61%
2050850-2074701	questionable	16	NC_009904	37.26%

Table S2 General features of the CRISPR loci identified in the *L. helveticus* KLDS1.8701 genome.

Position	Length	Repeat consensus	Repeat length	Spacers number
735863-736129	266 bp	GTTTTCTCCACGTAAGTGGAGGT	23 bp	4
890302-891796	1494 bp	GTATTCTCCACGTATGTGGAGGTGATCC	28 bp	24
2050850-2074701	759 bp	GTATTCTCCACGTATGTGGAGGTGATCC	28 bp	12

Table S3 Putative genes for Type I restriction modification (R/M) system in the *L. helveticus* KLDS1.8701 genome.

Encoded protein	Locus tag(s)
Type I restriction enzyme, R subunit	HUO_RS03985
Type I restriction enzyme M protein	HUO_RS01105
	HUO_RS03980
	HUO_RS03990
	HUO_RS04630
	HUO_RS06070
	HUO_RS08260
Type I restriction enzyme, S subunit	HUO_RS01110
	HUO_RS04635

Table S4 Putative antibiotics resistance genes in the *L. helveticus* KLDS1.8701 genome by searching with the comprehensive antibiotic resistance database (CARD).

Locus	ARO	Associate antibiotics	E-value	Identity	Coverage
HUO_RS00060	ARO:3004045	isoniazid	5.52E-82	47	98
HUO_RS00090	ARO:3003463	isoniazid	6.02E-78	36	92
HUO_RS00235	ARO:3000535	efflux pump complex	8.89E-18	30	74
HUO_RS00240	ARO:3002881	lincosamide	3.28E-20	30	80
HUO_RS00350	ARO:3002832	efflux pump complex	1.94E-24	32	83
HUO_RS00355	ARO:3002833	efflux pump complex	2.40E-18	34	79
HUO_RS00460	ARO:3000535	efflux pump complex	1.48E-52	42	92
HUO_RS00790	ARO:3000124	beta-lactam	1.61E-18	30	79
HUO_RS00875	ARO:3003459	fluoroquinolone	0	47	97
HUO_RS01135	ARO:3000535	efflux pump complex	9.88E-29	32	100
HUO_RS01270	ARO:3002943	vancomycin	3.28E-57	35	83
HUO_RS01440	ARO:3002928	vancomycin	5.92E-58	43	95
HUO_RS01580	ARO:3000535	efflux pump complex	4.61E-29	32	88
HUO_RS01705	ARO:3000535	efflux pump complex	1.19E-39	37	87

HUO_RS01715	ARO:3002909	vancomycin	7.76E-69	36	95
HUO_RS01780	ARO:3000535	efflux pump complex	5.46E-36	38	91
HUO_RS02075	ARO:3003730	mupirocin	1.30E-07	30	70
HUO_RS02165	ARO:3003775	fosfomycin	7.56E-98	40	99
HUO_RS02325	ARO:3002972	vancomycin	1.48E-46	31	83
HUO_RS02630	ARO:3003950	efflux pump complex	2.46E-25	31	84
HUO_RS02635	ARO:3003748	efflux pump complex	2.72E-27	30	87
HUO_RS03565	ARO:3003730	mupirocin	1.15E-09	32	78
HUO_RS03870	ARO:3002987	efflux pump complex	3.25E-30	30	99
HUO_RS04155	ARO:3000838	efflux pump complex	1.00E-80	51	97
HUO_RS04160	ARO:3000839	efflux pump complex	1.06E-74	34	80
HUO_RS04895	ARO:3002818	efflux pump complex	3.08E-37	49	86
HUO_RS05070	ARO:3002985	polymyxin	2.61E-44	35	80
HUO_RS05975	ARO:3003294	fluoroquinolone	2.39E-157	35	96
HUO_RS05980	ARO:3003459	fluoroquinolone	0	44	96
HUO_RS06105	ARO:3000535	efflux pump complex	3.52E-34	34	89
HUO_RS06510	ARO:3000300	dalfopristin	4.42E-157	45	99
HUO_RS07130	ARO:3003950	efflux pump complex	1.25E-25	31	78
HUO_RS07330	ARO:3002857	trimethoprim	5.05E-33	34	95
HUO_RS07575	ARO:3003359	elfamycin	0	68	98
HUO_RS07860	ARO:3003578	polymyxin	7.76E-43	31	99
HUO_RS08210	ARO:3002963	vancomycin	1.44E-13	31	86
HUO_RS08230	ARO:3002925	vancomycin	4.27E-54	40	97
HUO_RS09145	ARO:3000535	efflux pump complex	8.14E-42	36	90
HUO_RS09155	ARO:3002882	lincosamide	0	48	99
HUO_RS09160	ARO:3002881	lincosamide	0	48	96
HUO_RS09940	ARO:3000535	efflux pump complex	5.28E-35	36	97
HUO_RS09970	ARO:3000535	efflux pump complex	6.79E-51	41	86
HUO_RS10220	ARO:3003986	efflux pump complex	2.40E-72	30	99
HUO_RS10270	ARO:3002987	efflux pump complex	3.29E-22	31	96
HUO_RS10320	ARO:3000575	vancomycin	9.78E-08	33	78
HUO_RS10355	ARO:3002925	vancomycin	1.96E-68	48	99
HUO_RS10360	ARO:3002882	lincosamide	3.51E-106	34	86
HUO_RS10365	ARO:3002881	lincosamide	1.89E-108	34	90
HUO_RS10400	ARO:3003470	aminoglycoside	1.97E-24	30	77
HUO_RS10460	ARO:3000535	efflux pump complex	3.51E-46	39	98

Table S5 Putative virulence factors in the *L. helveticus* KLDS1.8701 genome by searching with the virulence factor database (VFDB).

Locus tag(s)	VFDB_ID	VFDB_Genes
HUO_RS03090	VFG012103	(groEL) chaperonin GroEL
HUO_RS01440	VFG006826	(lisR) two-component response regulator
HUO_RS02290	VFG005363	(sspA) surface protein D
HUO_RS06430	VFG037112	(msrA/B(pilB)) peptide methionine sulfoxide reductase msrA/msrB
HUO_RS05430	VFG043573	(CT396) molecular chaperone DnaK
HUO_RS05615	VFG026967	(sigA/rpoV) RNA polymerase sigma factor rpoD
HUO_RS02030	VFG005801	(alp2) alpha-like protein
HUO_RS07575	VFG016490	(tuf) translation elongation factor Tu
HUO_RS08570	VFG000077	(clpP) ATP-dependent Clp protease proteolytic subunit
HUO_RS06800	VFG000080	(clpE) ATP-dependent protease
HUO_RS08890	VFG005871	(hasC) UTP--glucose-1-phosphate uridylyltransferase
HUO_RS10015	VFG005865	(SMU.322c) glucose-1-phosphate uridylyltransferase

Table S6 Body weight and food data of male and female rats after oral administration of *L. helveticus* KLDS1.8701 for 28 days.

Index	Males			Females		
Treatment	Control	Low	High	Control	Low	High
Body weight gain (g)	69.9±6.7	72.6±7.1	67.1±6.1	32.0±2.7	30.5±2.1	28.4±3.1
Daily Food consumption (g)	24.1±0.9	23.7±1.3	23.1±1.8	16.9±1.2	16.8±1.1	16.5±1.7
Food Efficiency	0.104±0.011	0.109±0.007	0.101±0.003	0.068±0.006	0.069±0.006	0.065±0.008

Values are presented as mean ± standard deviation (n=6). Control, sterile normal saline; Low, 1 × 10^9 CFU of *L. helveticus* KLDS1.8701/kg BW; High, 1 × 10^{10} CFU of *L. helveticus* KLDS1.8701/kg BW.

Table S7 Relative organ weights (%) of male and female rats after oral administration of *L. helveticus* KLDS1.8701 for 28 days.

Organs	Males			Females		
Treatment	Control	Low	High	Control	Low	High
Heart	0.335±0.038	0.300±0.049	0.321±0.136	0.307±0.033	0.299±0.027	0.296±0.002
Liver	2.898±0.361	2.931±0.254	3.055±0.965	3.067±0.277	3.489±0.580	2.865±0.277
Spleen	0.164±0.047	0.178±0.029	0.183±0.036	0.204±0.052	0.195±0.040	0.215±0.030
Lung	0.796±0.143	0.811±0.162	0.841±0.078	0.636±0.092	0.640±0.163	0.669±0.058
Kidney	0.368±0.032	0.332±0.018	0.341±0.044	0.313±0.026	0.308±0.030	0.302±0.017
Brain	0.562±0.037	0.610±0.052	0.591±0.033	0.720±0.065	0.701±0.043	0.713±0.056
Adrenal	0.021±0.004	0.019±0.002	0.018±0.004	0.028±0.002	0.031±0.004	0.031±0.003
Thymus	0.027±0.007	0.029±0.017	0.031±0.005	0.023±0.007	0.021±0.004	0.020±0.011
Testes	0.476±0.052	0.480±0.021	0.468±0.085	-	-	-
Epididymides	0.322±0.017	0.328±0.053	0.341±0.040	-	-	-
Uterus	-	-	-	0.242±0.053	0.250±0.041	0.237±0.045
Ovary	-	-	-	0.062±0.007	0.053±0.006	0.055±0.009

Values are presented as mean ± standard deviation (n=6).Control, sterile normal saline; Low, 1 × 10^9 CFU of *L. helveticus* KLDS1.8701/kg BW; High, 1 × 10^{10} CFU of *L. helveticus* KLDS1.8701/kg BW.

Table S8 β-glucosidase and β-glucuronidase activities in the cecal contents of male and female rats after oral administration of *L. helveticus* KLDS1.8701 for 28 days.

**Treatment**	**Males**		**Females**	
	β-glucosidase(unit/g)	β-glucuronidase (unit/g)	β-glucosidase(unit/g)	β-glucuronidase (unit/g)
Control	9.40±0.53	3.69±0.39[a]	9.66±0.51	3.73±0.42[a]
Low	9.16±0.45	2.56±0.21[b]	9.48±0.49	2.72±0.28[b]
High	9.27±0.86	2.58±0.39[b]	9.75±0.66	2.59±0.52[b]

Values are presented as mean ± standard deviation (n=6). Significant differences ($P < 0.05$) among different treatments are indicated with different letters (a, b). Control, sterile normal saline; Low, 1 × 10^9 CFU of *L. helveticus* KLDS1.8701/kg BW; High, 1 × 10^{10} CFU of *L. helveticus* KLDS1.8701/kg BW.
